# Exposure to 17β-Oestradiol Induces Oxidative Stress in the Non-Oestrogen Receptor Invertebrate Species *Eisenia fetida*

**DOI:** 10.1371/journal.pone.0145426

**Published:** 2015-12-22

**Authors:** Zbynek Heger, Petr Michalek, Roman Guran, Barbora Havelkova, Marketa Kominkova, Natalia Cernei, Lukas Richtera, Miroslava Beklova, Vojtech Adam, Rene Kizek

**Affiliations:** 1 Department of Chemistry and Biochemistry, Mendel University in Brno, Zemedelska 1, CZ-613 00 Brno, Czech Republic, European Union; 2 Central European Institute of Technology, Brno University of Technology, Technicka 3058/10, CZ-616 00 Brno, Czech Republic, European Union; 3 Department of Ecology and Diseases of Game, Fish and Bees, Faculty of Veterinary Hygiene and Ecology, University of Veterinary and Pharmaceutical Sciences, Palackeho 1–3, CZ-612 42 Brno, Czech Republic, European Union; Jinling Institute of Technology, CHINA

## Abstract

**Background:**

The environmental impacts of various substances on all levels of organisms are under investigation. Among these substances, endocrine-disrupting compounds (EDCs) present a threat, although the environmental significance of these compounds remains largely unknown. To shed some light on this field, we assessed the effects of 17β-oestradiol on the growth, reproduction and formation of free radicals in *Eisenia fetida*.

**Methodology/Principal Findings:**

Although the observed effects on growth and survival were relatively weak, a strong impact on reproduction was observed (50.70% inhibition in 100 μg/kg of E_2_). We further demonstrated that the exposure of the earthworm *Eisenia fetida* to a contaminant of emerging concern, 17β-oestradiol (E_2_), significantly affected the molecules involved in antioxidant defence. Exposure to E_2_ results in the production of reactive oxygen species (ROS) and the stimulation of antioxidant systems (metallothionein and reduced oxidized glutathione ratio) but not phytochelatins at both the mRNA and translated protein levels. Matrix-assisted laser desorption/ionization (MALDI)-imaging revealed the subcuticular bioaccumulation of oestradiol-3,4-quinone, altering the levels of local antioxidants in a time-dependent manner.

**Conclusions/Significance:**

The present study illustrates that although most invertebrates do not possess oestrogen receptors, these organisms can be affected by oestrogen hormones, likely reflecting free diffusion into the cellular microenvironment with subsequent degradation to molecules that undergo redox cycling, producing ROS, thereby increasing environmental contamination that also perilously affects keystone animals, forming lower trophic levels.

## Introduction

Contaminants of emerging concern such as endocrine-disrupting compounds (EDCs), which are of an organic nature, have been detected in wastewater, surface water, and drinking water worldwide [1–3]. EDCs enter the wastewater treatment system from the excreta of livestock and humans [4]. These substances can be natural oestrogens—17β-oestradiol (E_2_), oestrone (E_1_) or oestriol (E_3_) and also of synthetic origin, like 17α-ethynyloestradiol (EE_2_), a common ingredient of human contraceptives. All species, sexes, and classes of farm animals eliminate natural oestrogenic hormones into the environment [5]; however, the environmental significance of this contamination remains largely unknown.

Under common conditions, E_2_ is expected to rapidly dissipate in the soil, reflecting absorption [6]. Subsequently, E_2_ can be transformed into E_1_, which persists in the soil for a long time without further degradation, as previously described [7]. Alternatively, E_2_ is degraded through a metabolic oxidation sequence to produce E_3_ and E_1_ [8] and further transformed into semiquinones and quinones [9]. Although the potential fate of E_2_ in the soil system has been suggested, the actual biological impact of this substance on soil inhabitants is not fully understood.

The soil environment contains many organisms from different trophic levels. Earthworms are animals from the lower trophic level (60–80% of the total biomass), whose contamination can result in bioaccumulation and biomagnification along the food chain [10]. The semi-permeable body walls of these animals facilitate the absorption of various low-molecular weight chemicals. Earthworms are well known for their bioaccumulation of environmental pollutants both *via* the ingestion of contaminated organic matter and water and the body surface [11]. Thus, earthworms are commonly used as bioindicators for evaluating pollution in toxicity tests.

Although the earthworm *Eisenia fetida* is one of the recommended test species for environmental surveillance (together with *E*. *andrei*) [12], to the best of our knowledge, data concerning the effects of E_2_ on protective molecular mechanisms are unavailable. In a survey of annelid endocrine disruptors, Krajniak reported that EDCs affect invertebrates by inducing decreased growth and reproductive output, delayed sexual maturation and immune system inhibition. However, the mechanisms underlying these responses remain largely unknown [13].

Some studies have demonstrated that the exogenous addition of E_2_ increases oxidative stress in fish [14] or rat-derived cell lines [15], describing elevation of a number of indicators of oxidative stress, including metallothionein (MT) and glutathione peroxidase (GPx). Despite this fact, no data regarding this phenomenon in invertebrates are available. Considering the oxidative stress hypothesis, it is general fact that unnatural generation of free radicals alters growth and reproduction attributes, as was described in aquatic invertebrates [16]. However, involvement of estrogens in such alterations in soil invertebrates needs to be addressed.

Because the majority of E_2_ is absorbed in soil particles and subsequently transformed into other biologically active low-molecular weight products, thereby affecting soil inhabitants, we examined whether E_2_ exposure influences the substantial antioxidant and transport mechanisms in *E*. *fetida*. To address this question, we assessed the effects of E_2_ on the growth, reproduction and formation of free radicals in *E*. *fetida*. To obtain further insight into E_2_ influences, the effects on the expression of MT, phytochelatin synthase (PCS) and GPx as well as the subsequent detection of metallothionein, reduced and oxidized glutathiones (GSH and GSSG), GPx and phytochelatin isoforms (PC_2_, PC_3_ and PC_4_, respectively) at the translated protein/peptide level was revealed.

## Results and Discussion

### The effects of E_2_ exposure on the growth and reproduction of *E*. *fetida*

The application of manure to a field results in the surface runoff of E_2_, reaching dozens of μg per litre; however these numbers depend on the application rate and time between applications [17]. Thus, for these experiments, we utilized the concentration range of 0–100 μg/kg. The entire experiment fulfilled the OECD validity criteria, suggesting that each replicate (containing 10 adults) should produce ≥ 30 juveniles through the end of the experimental period; the coefficient of variation of reproduction should be ≤ 30%; and the adult mortality over the initial 4 weeks of the experimental period should be ≤ 10%. The results demonstrated that the highest applied concentrations of E_2_ (100 μg/kg) induced 10% *E*. *fetida* mortality after 4 weeks of exposure (Table 1). Although the observed effects on growth (biomass) and survival were weak, a strong impact on reproduction was observed (50.70% inhibition in 100 μg/kg of E_2_).

**Table 1:** Summary of the toxic effects of E_2_ on *E*. *fetida* growth and reproduction.

Concentration of E_2_ (μg/kg)	No. of adults		Biomass			Mortality (%)	No. of juveniles produced (mean ± SD)	CV (%)**	Inhibition of reproduction (%)
	0^th^ week	4^th^ week	0^th^ week (mean ± SD)* (g)	4^th^ week(mean ± SD) (g)	Increase (mean ± SD) (g)				
**0 (Control)**	10	10	4.15 ± 0.41	6.12 ± 0.20	1.97 ± 0.11	0	143.3 ± 32.9	23	0.00
**10**	10	10	4.13 ± 0.25	6.20 ± 0.41	2.07 ± 0.15	0	158.0 ± 53.2	24	-10.23
**30**	10	10	4.15 ± 0.34	6.09 ± 0.24	1.94 ± 0.08	0	168.3 ± 59.4	26	-15.35
**50**	10	10	4.16 ± 0.16	6.06 ± 0.39	1.90 ± 0.12	0	177.3 ± 28.6	16	-23.72
**80**	10	10	4.20 ± 0.22	6.04 ± 0.42	1.84 ± 0.10	0	107.3 ± 20.0	18	25.12
**100**	10	9	4.10 ± 0.15	5.50 ± 0.23	1.40 ± 0.06	10	70.7 ± 6.9	10	50.70

*SD—standard deviation;

**CV—coefficient of variation

**Fig 1:**
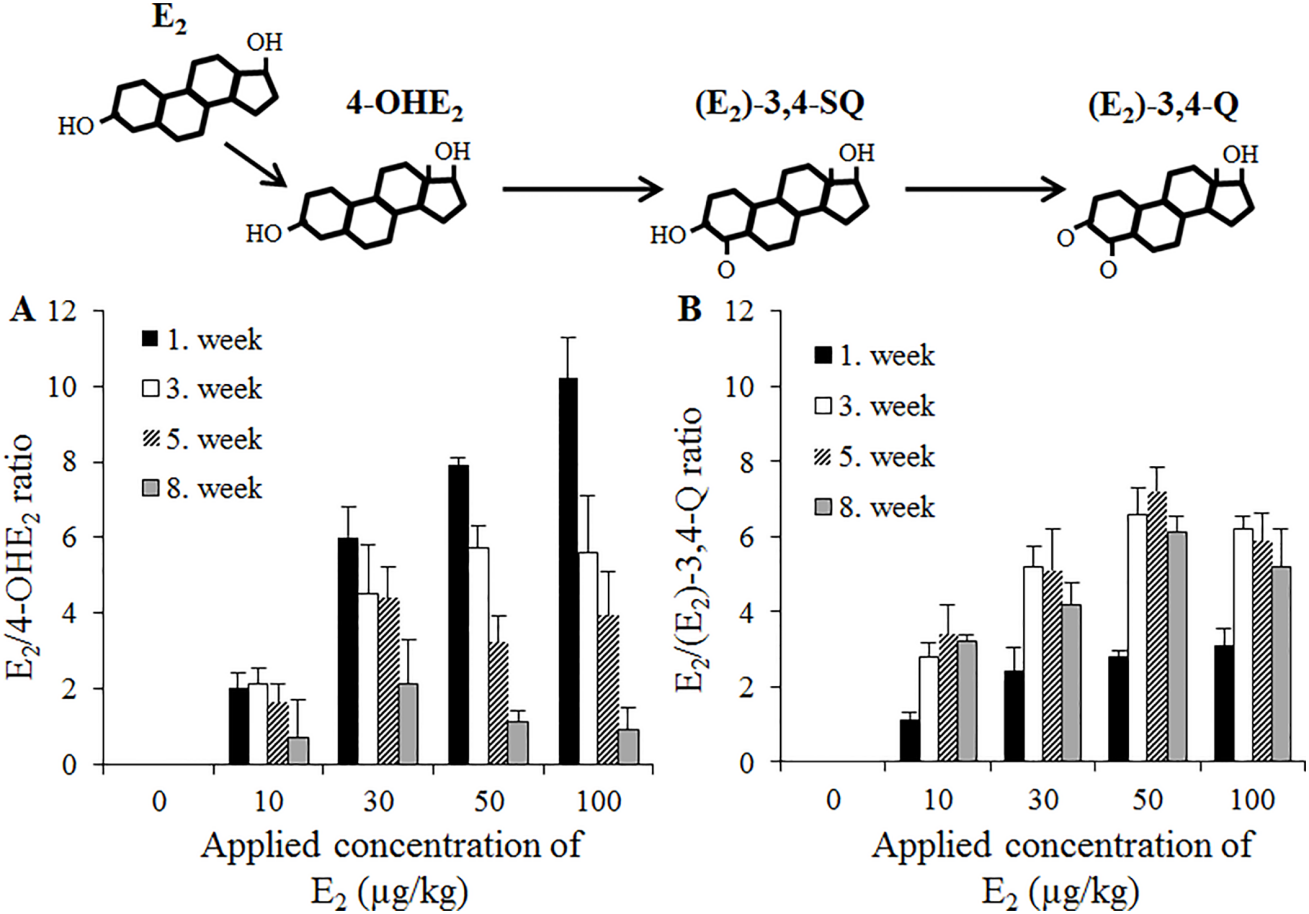
Schematic depiction of E_2_ metabolites. The E_2_ ratios to (**A**) 4-OHE_2_ and (**B**) the final metabolite (E_2_)-3,4-Q represents the conversions over time. In control samples, no E_2_, 4-OHE_2_ or (E_2_)-3,4-Q was found. To evaluate the conversion ratios, the following mass weights were utilized: [E_2_ + H]^+^ *m/z* 273.38 Da, [4-OHE_2_ + H]^+^ *m/z* 289.38 Da and [(E_2_)-3,4-Q + H]^+^ at *m/z* 287.36 Da. The values are presented as the means of three independent replicates (*n* = 3). The vertical bars indicate standard error.

**Fig 2:**
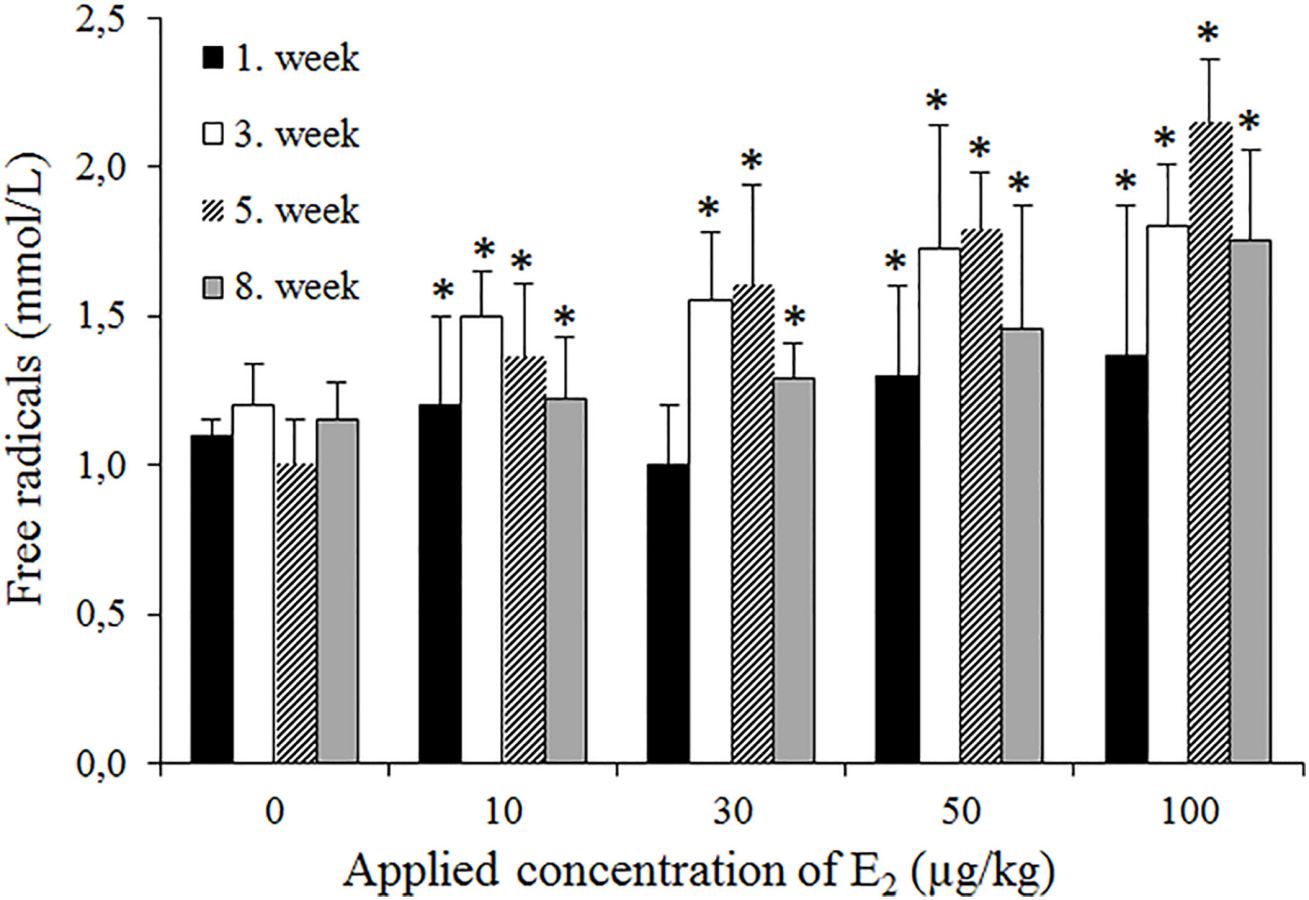
The free radical contents in *E*. *fetida* immediately after the termination of the test. The error bars indicate standard deviations. The asterisks indicate significant differences (*p*<0.05) compared with the control groups.

Notably, lower E_2_ doses (10, 30 and 50 μg/kg) stimulated reproduction (expressed as the negative inhibition of reproduction at -10.2; -15.4 and -23.7%, respectively). However, higher concentrations (80 and 100 μg/kg) significantly inhibited reproduction (25.1 and 50.7%), with an EC_50_ of 98 μg/kg for E_2_. According to Marksman and colleagues, earthworms bioaccumulate E_2_ and other EDCs [18], however the impact of the animals on biologically important molecules remains elusive. Thus, we aimed to determine whether the MALDI-TOF profiling of tissue homogenates would reveal the presence of E_2_ in earthworm homogenates. Fig 1A demonstrates that there are no E_2_ peaks (272.38 Da) in the untreated control; however, treatment resulted in the formation of peaks corresponding to 4-hydroxyoestradiol (4-OHE_2_) and the final metabolic product of E_2_, oestradiol-3,4-quinone (E_2_)-3,4-Q) (Fig 1B), which is frequently associated with oxidative stress [19]. Thus, we further evaluated the formation of free radicals, and the results of the chlorophyllin assay revealed a positive correlation between the E_2_ dose and the amount of free radicals (Fig 2). Although E_2_ inhibits oxidative stress and prevents H_2_O_2_-induced apoptosis in some mammalian cells [20], other studies have demonstrated that the exogenous addition of E_2_ elevates oxidative stress in fish [14] or rat-derived cell lines [15]. This result likely reflects the degradation of E_2_ to 4-OHE_2_, which subsequently undergoes redox cycling, producing reactive oxygen species (ROS) [19]. ROS affect the cellular microenvironment in a concentration-dependent manner and influence stimulatory or inhibitory growth signals. Thus, we further described the potential antioxidant protection in E_2_-exposed *E*. *fetida* individuals.

### The effects of E_2_ exposure on gene expression

**Fig 3:**
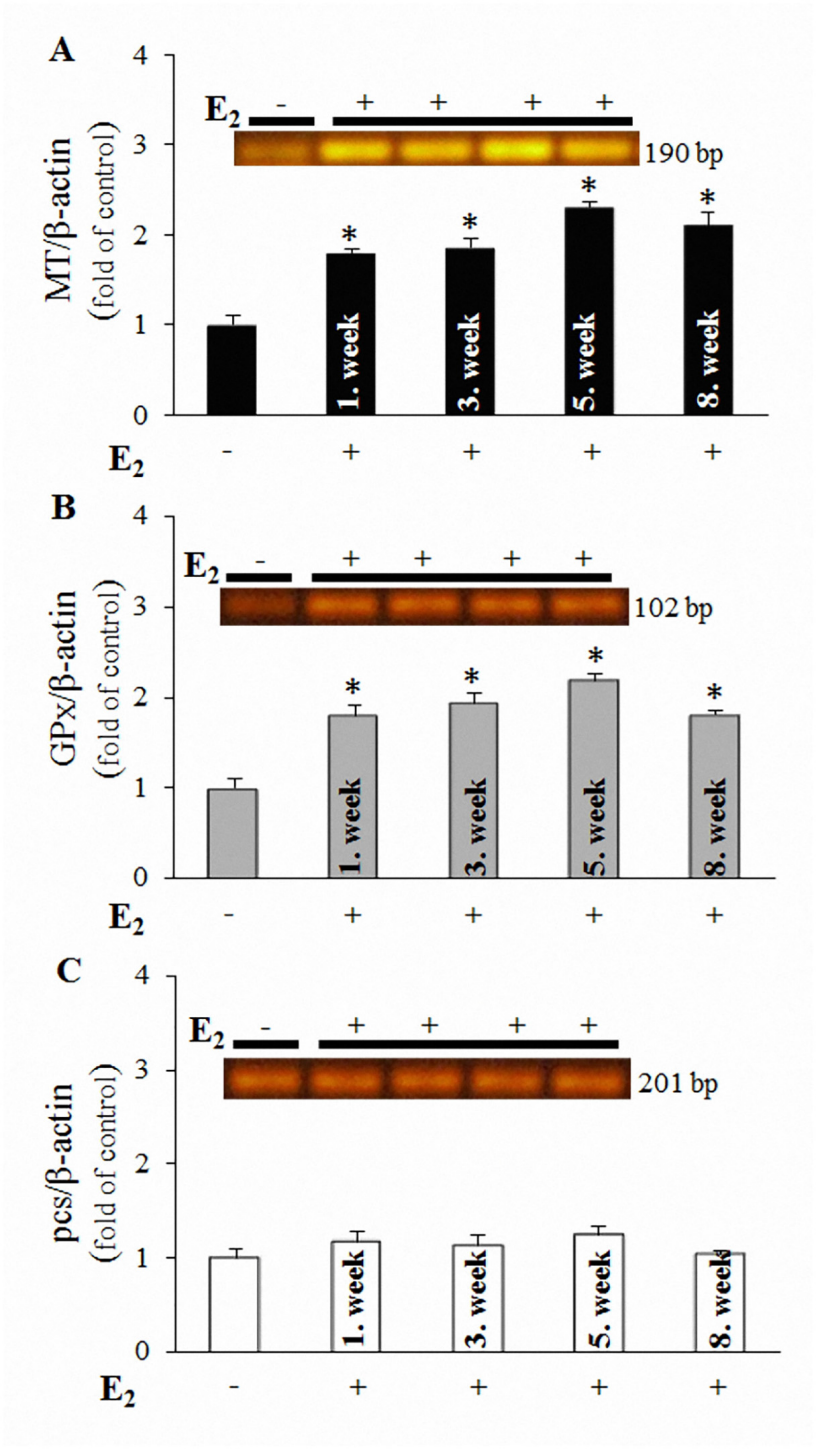
Influence of E_2_ exposure (100 μg/kg) on the expression of mRNA encoding selected antioxidant molecules. (**A**) *MT*, (**B**) *GPx* and (**C**) *pcs* genes in *E*. *fetida*, collected in the 1^st^; 3^rd^; 5^th^ and 8^th^ weeks of the experiment. For evaluation, the corresponding molecular-weight bands of amplicons coinciding with the values shown in Table 1 were obtained using RT-PCR. The fluorescence intensities of the bands, obtained using ethidium bromide staining, were transformed into numerical forms, and expression was normalized to *β-actin* expression from the same cDNA template. The values are presented as the means of three independent replicates (*n* = 3). The vertical bars indicate standard error. The asterisks indicate significant differences (*p* < .05) compared with the control groups.

We examined the expression of three genes involved in oxidative stress (*MT*, *GPx* and *pcs*). *MT* expression is characterized by strong induction associated with E_2_ exposure (Fig 3A). Yoshido Kadota and coworkers demonstrated that the level of metallothionein gene expression directly determines the ROS scavenging of MT [21], and it is obvious that exposure to E_2_ induces oxidative conditions in *E*. *fetida*. The analysis of *GPx* mRNA expression revealed that E_2_ exposure up-regulates this gene (Fig 3B). GPx converts H_2_O_2_ to H_2_O with the subsequent oxidation of GSH to GSSG [22]. Although GSH acts as a substrate for phytochelatins and metal exposure results in the depletion of glutathiones as a result of elevated synthesis [23], intracellular ROS did not affect the expression of *pcs* mRNA (Fig 3C). These data suggest divergent roles of closely linked antioxidant mechanisms. As previously described, *pcs* is up-regulated during cellular interactions with metal ions, but based on the results of the present study, this gene is not up-regulated in response to ROS derived from the metabolic degradation of E_2_.

### The effects of exposure to E_2_ on the levels of antioxidant molecules

**Fig 4:**
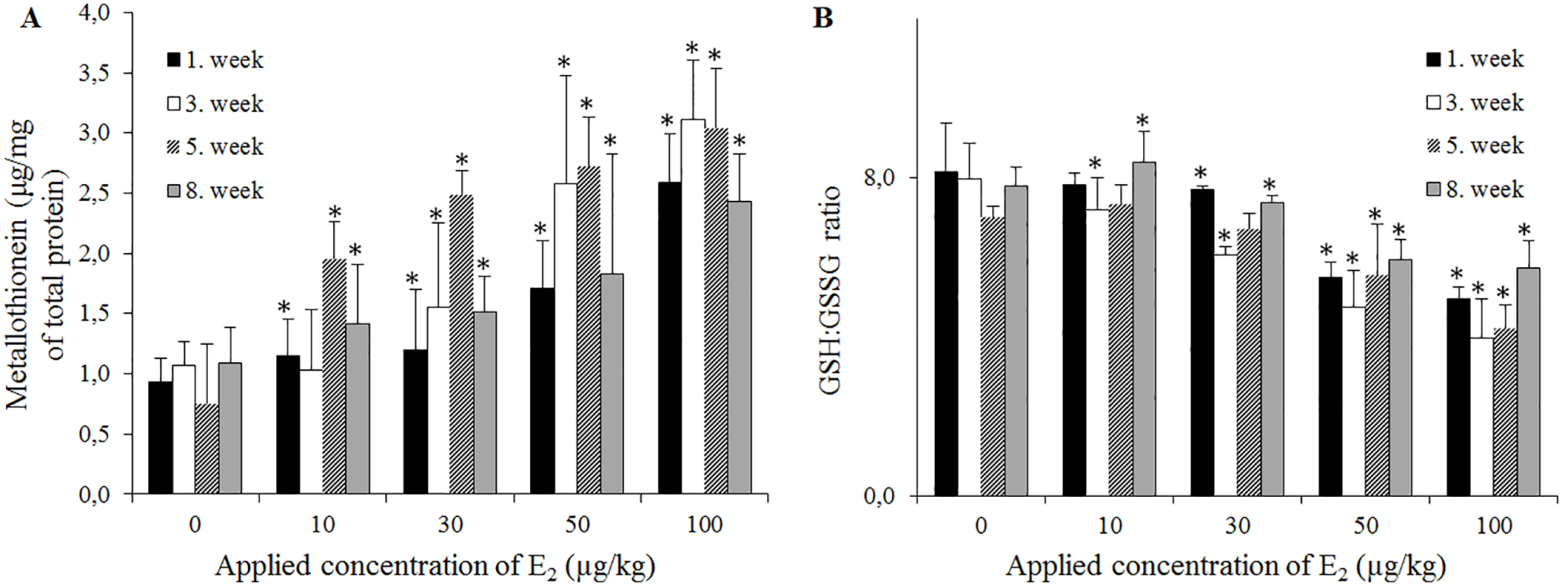
The levels of antioxidant molecules in *E*. *fetida* after exposure to E_2_ (0–100 μg/L). (**A**) The levels of metallothionein, determined using DPV. (**B**) The ratio between GSH and GSSG, determined using HPLC-ED. The antioxidant molecules were analysed in the 1^st^; 3^rd^; 5^th^ and 8^th^ weeks of the experiment. The values are presented as the means of three independent replicates (*n* = 3). The vertical bars indicate standard errors. The asterisks indicate significant differences (*p*.05) compared with the control groups.

Antioxidant defence involves two general classes of molecules, including enzymes (SOD, CAT, etc.) and water-soluble reductants, such as GSH, MT or PCs. Based on previous experience, we selected these biochemical parameters based on susceptive compensatory responses to various environmental contaminants (reviewed in [24]). MTs are a family of evolutionarily conserved proteins that are generally involved in the intracellular binding of metals, and a role for these proteins has been described in *E*. *fetida* after exposure to Zn, Cu, Pb, and Cd, respectively [25]. Another important function of MTs is protection against free radical attacks. MTs scavenge a variety of ROS molecules, reflecting the cysteinyl thiol moieties of this molecule [26]. Fig 4A illustrates the response of MT formation to treatment with E_2_ and suggests a dose-dependent effect, with a significant increase in MT production compared with the untreated control. The highest MT values were achieved in the 3^rd^ and 5^th^ sampling weeks (3.11 and 3.04 μg/mg of total protein). Further analyses of the samples were conducted in the 8^th^ week, revealing a significant decrease in MT levels, which reflects a return to redox equilibrium. Previous studies have shown that MT expression is primarily controlled at the transcription level (consistent with the results of the present study) and affected through a variety of environmental stressors [27]. The obtained data demonstrated that E_2_ induces MT expression; however E_2_ induces MT less effectively than metal ions [28].

**Fig 5:**
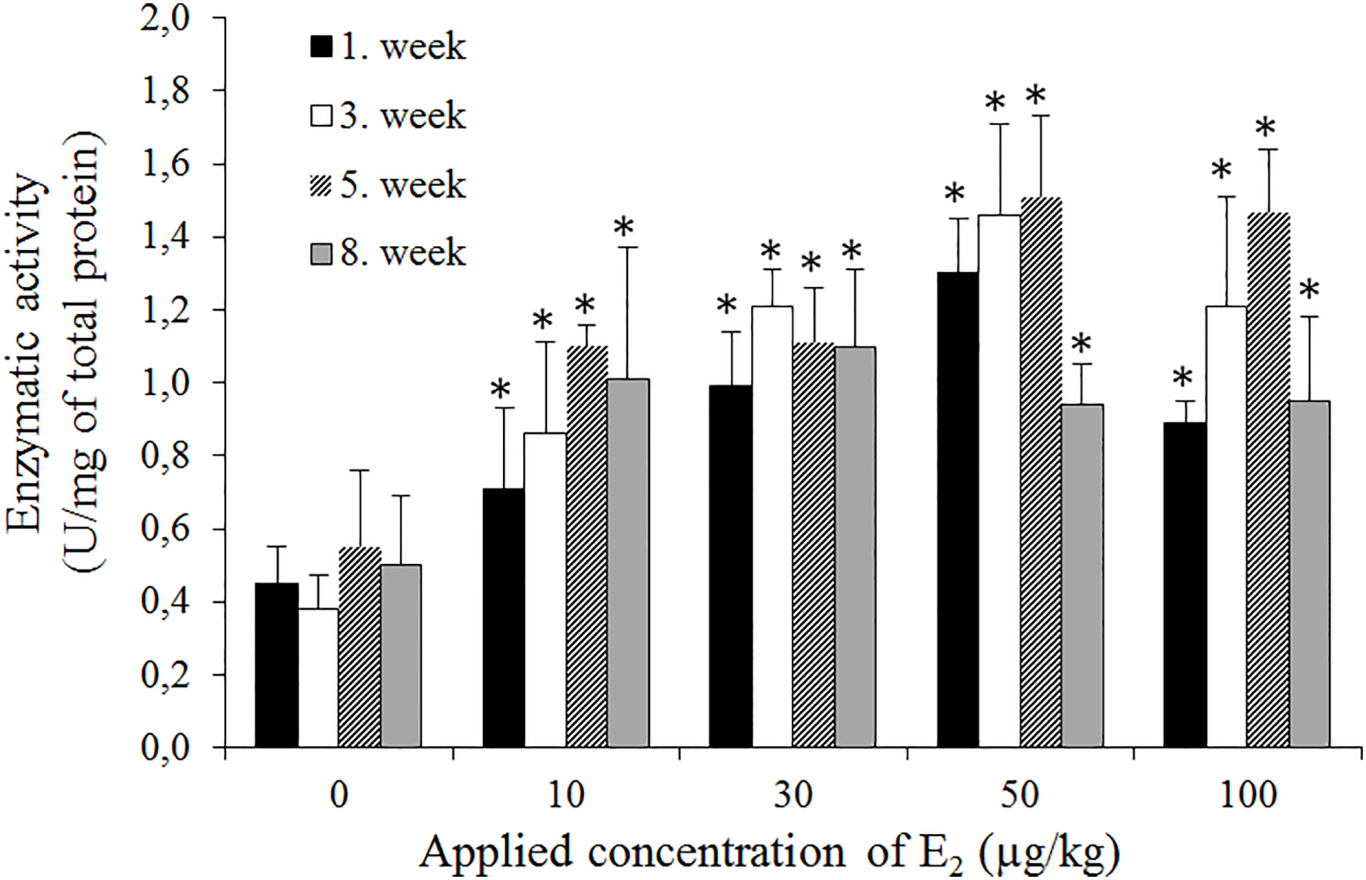
Determination of GPx activity after exposure to E2 (0–100 μg/kg). The enzymatic activity was examined in the 1^st^; 3^rd^; 5^th^ and 8^th^ weeks of the experiment. The values are presented as the means of three independent replicates (*n* = 3). The vertical bars indicate the standard error. The asterisks indicate significant differences (*p* < .05) compared with the control groups.

GSH is the most abundant antioxidant in aerobic cells, acting as a fundamental component of signalling processes, detoxifying certain xenobiotics and heavy metals [29]. Upon oxidative stress, GPx converts GSH to its oxidized form (GSSG). Thus, the GSH/GSSG ratio might serve as a marker of cellular stress [22]. Fig 4B demonstrates that the increasing E_2_ concentrations triggered a higher GSH-to-GSSG conversion, and similar to MT, the highest impact was observed in the 3^nd^ and 5^th^ weeks (4.0 and 4.2, respectively). Because the thiol moieties of GSH donate a reducing equivalent to other unstable molecules, such as ROS, and become reactive, readily forming GSSG, the significant decreasing GSH/GSSG ratio indicates oxidative stress in the *E*. *fetida* cellular microenvironment upon exposure to E_2_. Furthermore, we determined the enzymatic activity of GPx, and Fig 5 illustrates the elevation of activity during E_2_ exposure, consistent with the levels of *GPx* transcripts. Thus, it is obvious that the glutathione pathway is involved in defence against E_2_ metabolites.

**Fig 6:**
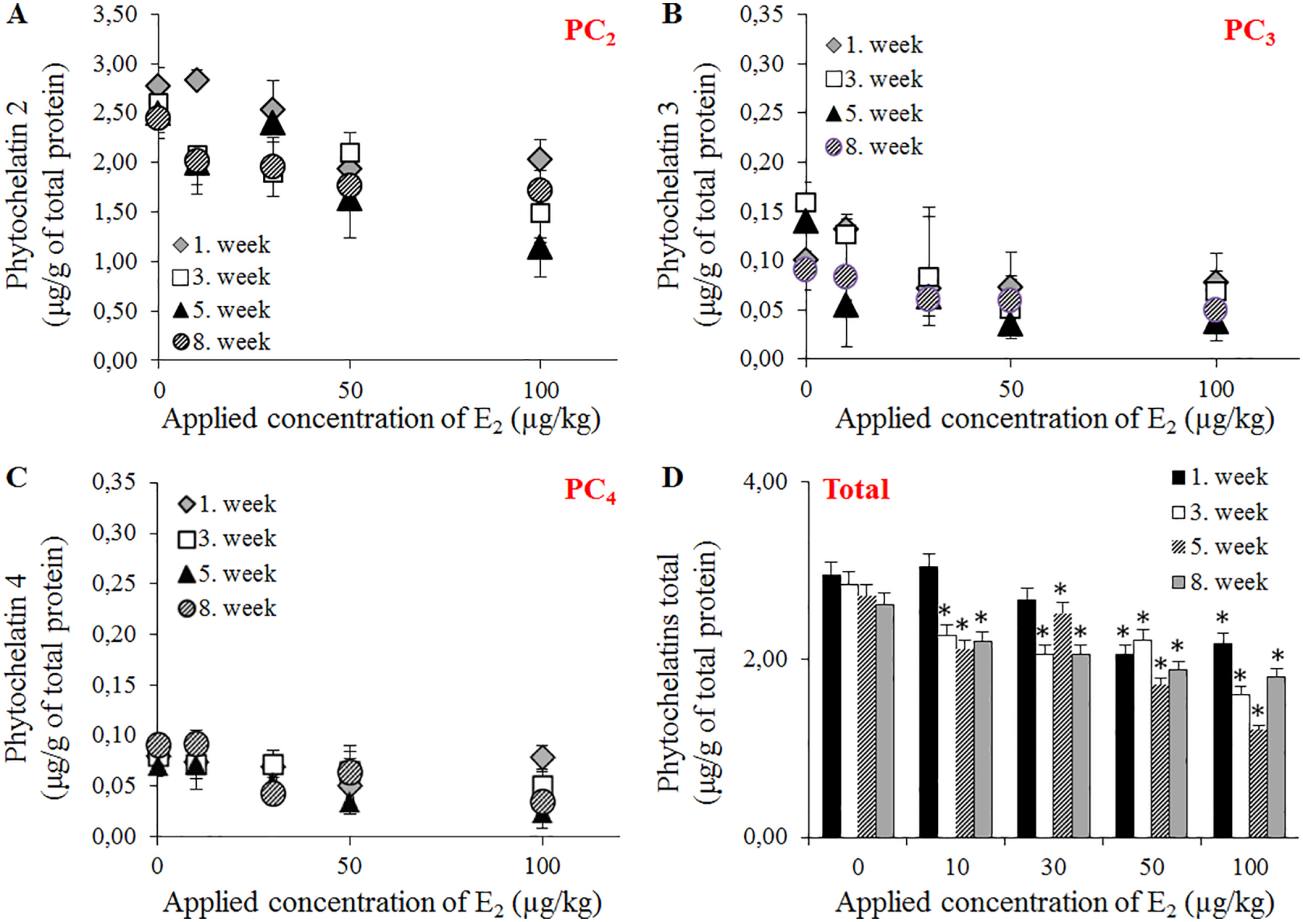
Expression of phytochelatin isoforms in *E*. *fetida* after exposure to E_2_ (0–100 μg/L) determined using HPLC-ED. (**A**) The levels of isoform PC_2_, (**B**) isoform PC_3_, (**C**) isoform PC_4_ and (**D**) all analysed isoforms. Other known isoforms (PC_5_, PC_6_) were not determined. The PCs were analysed in the 1^st^; 3^rd^; 5^th^ and 8^th^ weeks of the experiment. The values are presented as the means of three independent replicates (*n* = 3). The vertical bars indicate standard errors. The asterisks indicate significant differences (*p* < .05) compared with the control groups.

PCs are post-transcriptionally synthesized through constitutive phytochelatin synthase using GSH as a substrate. PCs play major roles in the detoxification of heavy metal ions in plants; however, the presence of PCs was also observed in *Caenorhabditis elegans* [30] and *Lumbricus rubellus* [31]. Although Brulle and coworkers determined the complete coding sequence of the *pcs* gene in *E*. *fetida* [32], the presence of PCs has not yet been described. Three isoforms (PC_2_, PC_3_ and PC_4_) were identified in the present study, and the biosynthesis of these compounds was negatively affected after E_2_ exposure. PC_2_ is a predominant isoform (the highest tissue concentration was 2.83 μg/g of total protein) in *E*. *fetida* (Fig 6A), followed by PC_3_ (0.16 μg/g of total protein) (Fig 6B) and PC_4_ (0.09 μg/g of total protein) (Fig 6C). The total PCs protein levels reflect decreasing PCs levels with elevated E_2_ concentration (Fig 6D). This phenomenon likely reflects a decline in GSH content, which is preferentially converted to GSSG. This conversion is also supported by the above-mentioned data, illustrating the largest decrease in the GSH/GSSG ratio in the 3^nd^ and 5^th^ weeks of exposure. Taken together, these results confirmed the presence of the *pcs* gene in *E*. *fetida*. Furthermore, evidence of the presence of PC isoforms in other metazoan species was obtained; however, these were not directly affected after E_2_ exposure but rather were more affected by the oxidation of the substrate glutathione. To elucidate the role of these peptides in the antioxidant protection of *E*. *fetida*, additional studies examining the response of PCs to the primary target, heavy metal ions, are needed.

**Fig 7:**
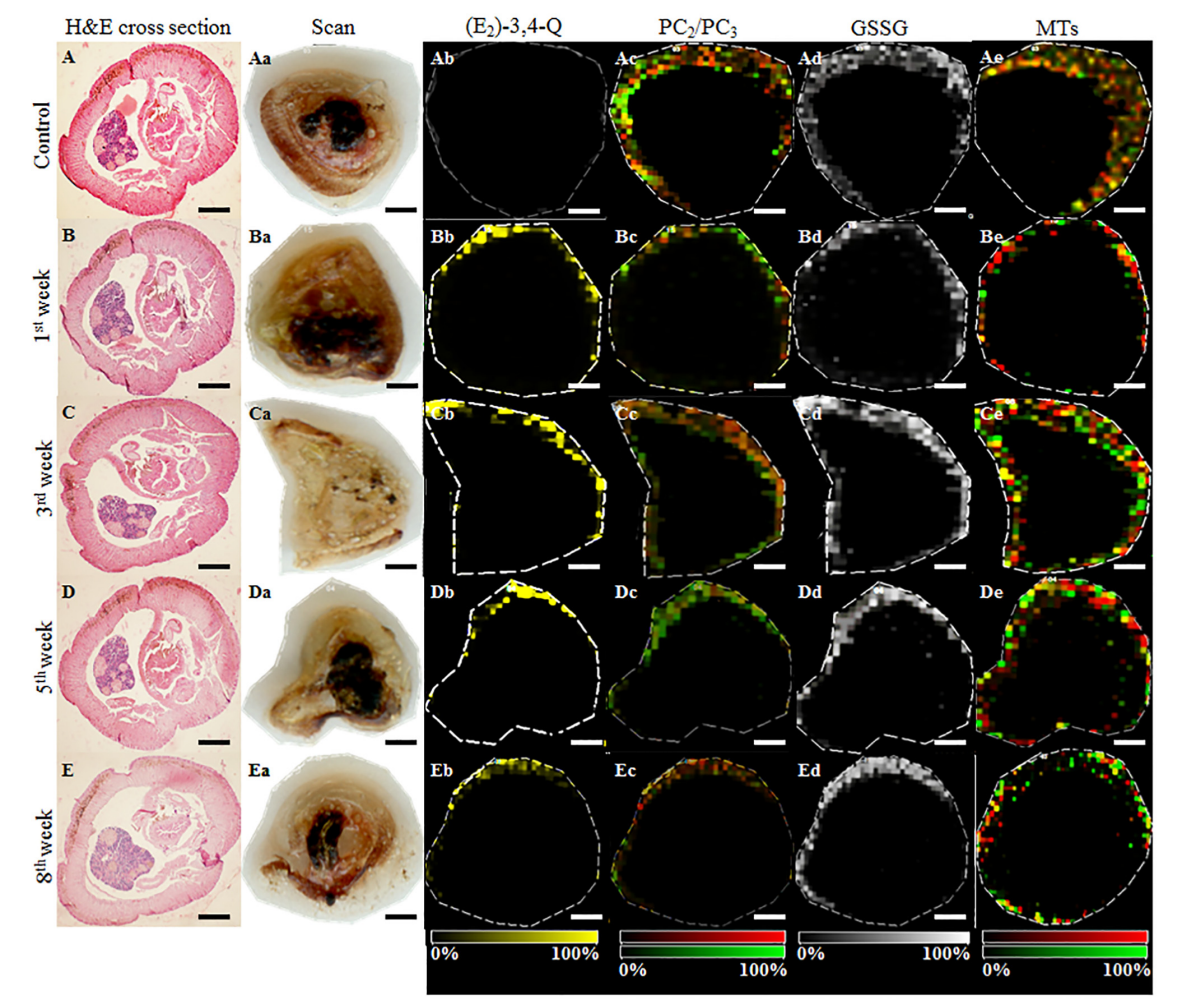
Examination of *E*. *fetida* cross sections after E_2_ exposure. H&E-stained cross sections of *E*. *fetida* exposed to E_2_ (100 μg/L), collected in the (**A**) 0^th^ (control), (**B**) 1^st^, (**C**) 3^rd^, (**D**) 5^th^ and (**E**) 8^th^ weeks of the experiment. The collected individuals were scanned (**a**) and employed for the MALDI-IMS analysis of (**b**) the final metabolites of E_2_—[(E_2_)-3,4-Q + H]^+^ at *m/z* 287.36 Da ± 0.05%, (**c**) [PC_2_ and PC_3_+ H]^+^ merge at *m/z* 541.61 Da ± 0.05% (red) and 773.91 Da ± 0.05% (green), respectively, (**d**) [GSSG + H]^+^ at *m/z* 613.64 Da ± 0.05% and (**e**) [MT_1_ + H]^+^ at *m/z* 4798.00 Da ± 0.05% (red) and [MT_2_ + H]^+^ at m/z 7412.01 Da (green). Matrix HCCA was used. The following conditions were used to acquire the MALDI spectra: 500 shots per raster spot, 45% laser energy and 50-μm spatial resolution. The length of scale bar is 500 μm.

To further demonstrate the impact of E_2_, cross sections of *E*. *fetida* individuals (Fig 7A–7E) collected in the 0^th^–8^th^ sampling weeks and the MALDI-IMS (Fig 7Aa–7Ee) data were examined. Although the histological sections revealed no significant impact on the earthworm organism, the MALDI-IMS data obtained from the tissue slides revealed that E_2_ exposure resulted in the subcuticular bioaccumulation of molecules with *m/z* 287.36 Da, corresponding to oestradiol-3,4-quinone quasi-molecular ions with hydrogen [(E_2_)-3,4-Q + H]^+^, whose presence was not determined in the untreated control (Fig 7b–7Eb). Hence, these sites were further utilized to examine antioxidant molecules. Particularly in the case of PC_2_/PC_3_, the decrease in intensity and abundance supported the HPLC-ED results. The PC_4_ isoform was not identified, likely reflecting the low amount and problematic ionization of this isoform. Similarly, the elevation of GSSG and MTs (two isoforms with *m/z* 4797.00 and 7411.01 Da, previously described in *E*. *fetida* [33]) was associated with the length of exposure.

### Proposed fate of E_2_ and its effects on the redox equilibrium in E. *fetida* cells

Based on the above-mentioned data, it is obvious that E_2_ exposure triggers a cascade of events, resulting in the elevation of biomarkers of oxidative stress. In vertebrates, E_2_ interacts with transmembrane oestrogen receptors (ERs), leading to translocation and signal transduction [34]. Because invertebrates do not possess ERs, with a few exceptions in which active ERs were detected only in *Platynereis dumerilii* and *Capitella capitata* [35], E_2_ can be internalized into the cellular microenvironment through free diffusion, which has been previously demonstrated for steroid hormones [36]. E_2_ undergoes degradation through physiological oxidation via enzymes from the superfamily of cytochrome P450 (CYPs), whose activity in *E*. *fetida* has previously been described [37]. It is likely that the activity of these enzymes results in the formation of 4-OHE_2_, which can subsequently be oxidized to oestradiol-3,4-semiquinone (E_2_)-3,4-SQ) and finally to (E_2_)-3,4-Q. These events are physiological and conditioned in the presence of E_2_ and CYPs enzymes [38]. (E_2_)-3,4-Q easily undergoes redox cycling, generating superoxide anions [19]. These products can further be converted to H_2_O_2_ through superoxide dismutase and H_2_O through GPx, which reversibly oxidizes two molecules of GSH to GSSG. As previously described, GSH is a fundamental substrate for the biosynthesis of PCs. Thus, the oxidation to GSSG decreases the formation of PC isoforms through phytochelatin synthase [31], whose gene expression is not affected after E_2_ exposure. In the case of MT, H_2_O_2_ is a major target of antioxidant activity, particularly reflecting the low reactivity of this molecule (other ROS are scavenged through GSH near the site of production), facilitating deeper penetration into the cell [39]. This interaction induces the oxidation of MT thiolate moieties, the loss of metal binding capacity, and the release of zinc from the physiologically predominant form, Zn-MT, and subsequent conversion to MT-disulphide. Selenium catalysts accelerate the reversible reduction process, resulting in the simultaneous formation of Zn-MT and oxidation of GSH to GSSG (which contributes to the decrease in the GSH:GSSG ratio, as shown in Fig 4B) [26]. Released zinc activates metal transcription factor 1 (MTF-1), which regulates the transcription of a wide array of genes, including *MT*. The induction of MT mRNA expression was higher after E_2_ exposure (Fig 3A), suggesting that ROS production in cells induced the release of zinc ions from metallothionein, leading to the induction of MT protein synthesis through MTF-1.

## Experimental Section

### Chemical compounds

All standards and other chemicals were purchased from Sigma-Aldrich (St. Louis, MO, USA) at ACS purity, unless otherwise noted.

### Tested earthworm species

The Oligochaete *Eisenia fetida* (family Lumbricidae) were obtained from the Ecotoxicological Laboratory of the University of Veterinary and Pharmaceutical Sciences, Brno, Czech Republic. Adult hermaphrodites between two and twelve months old (weighing between 350 and 500 mg), with fully developed clitellum, were used for testing.

### Reproduction test

The experiments were performed as described in OECD Guideline 222 [12]. The artificial soil used as a testing substrate was prepared as a mixture of 70% sand, 20% kaolin clay and 10% finely ground sphagnum peat, p*H* 6.0 ± 0.5, adjusted with CaCO_3_. All tests were conducted at 20 ± 2°C with a 16:8 (light/dark) photoperiod and illumination of 600 lx in the area of the test containers. Both, p*H* and moisture were evaluated at the beginning and end of the tests. During the test, the soil was contaminated with E_2_ dissolved in an adequate amount of deionized water to achieve soil moisture equal to 50% of the maximum water-holding capacity. The concentrations were selected according to an experimentally determined range. Each E_2_ stock solution was mixed with the soil immediately prior to use, generating nominal concentrations of 10, 30, 50, 80 and 100 μg/kg of E_2_ per vessel. Five replicates were used per tested concentration. The mortality and growth effects on adult worms were determined at the end of the 4^th^ week of exposure. The effects on reproduction were subsequently assessed after another 4 weeks after counting the number of offspring present in the soil. After the termination of the experiment, the earthworms were immediately washed with MilliQ water and bathed in 500 μL of RNA*later* ^®^ (Life Technologies, Carlsbad, CA, USA) to avoid undesired RNA degradation.

### Extraction and quantification of RNA

Each individual was crushed under liquid nitrogen using a mortar and pestle and subsequently mixed with 350 μL of Tissue Lysis Buffer (Roche, Basel, Switzerland) in an Eppendorf tube. After 30 min at 25°C, the samples were centrifuged (13000 × *g* at 20°C for 2 min) using an Eppendorf 5402 microcentrifuge (Eppendorf, Hamburg, Germany). Subsequently, 350 μL of the lysate supernatant was pipetted into the sample tube as a component of the MagNA Pure Compact RNA Isolation Kit (Roche, Basel, Switzerland). The isolation steps were performed according to the manufacturer’s instructions. The concentration of obtained RNA was quantified using Infinite M200 PRO (Tecan, Männedorf, Switzerland).

### Reverse transcription, PCR amplification and agarose gel electrophoresis

To obtain cDNA, 500 ng of total RNA was subjected to reverse transcription polymerase chain reaction (RT-PCR) using the High Capacity cDNA Reverse Transcription Kit (Life Technologies, Carlsbad, CA, USA) and random hexamer primers. The following reaction profile was used: 25°C for 10 min, 37°C for 120 min and 85°C for 5 min.

The obtained cDNA was diluted five times and subsequently used as a template for the amplification of *MT*, *pcs*, *GPx*, and *β-actin* gene fragments. The *Taq* polymerase chain reaction kit was purchased from New England BioLabs (Ipswich, MA, USA) and the PCR reaction mixture (25 μL) comprised 5 μL of cDNA, 1× standard *Taq* reaction buffer, 0.2 μM of each deoxynucleotide, 0.4 μM of each primer and 1 U of *Taq* DNA polymerase. The PCR reaction was performed using a Mastercycler Ep realplex4 (Eppendorf AG, Hamburg, Germany) with the following thermal profile: initial denaturation at 95°C for 4 min; 25 cycles of denaturation at 95°C for 30 s, annealing at 55°C for 30 s and extension at 72°C for 30 s; with the final extension at 72°C for 7 min. The DNA fragments were confirmed through electrophoresis on a 1.5% agarose gel. The intensity of the bands was quantified using Molecular Imaging software (Bruker Corporation, Billerica, MA, USA). The primers for PCR were synthesized using the synthesizer Expedite 8908 (Applied Biosystems, Waltham, MA, USA) with the sequences shown in Table 2.

**Table 2:** Sequences of the primers used for RT-PCR.

Gene	Abbreviation	GenBankAccession no.	Primer pair (5′-3′)*	Amplicon size (bp)
Beta-actin	*β-actin*	GU177854.1	TCCATCGTCCACAGAAAG	149
			AAATGTCCTCCGCAAGCT	
Metallothionein	*MT*	GU177855	CGCAAGAGAGGGATCAACTT	190
			ACAGCACCCCTTCTTGCAT	
Phytochelatin synthase	*pcs*	EF433776	ATGTCGTGCGGTTCATAACA	201
			TCTGCTTGATGGCGTACTTG	
Glutathione peroxidase	*GPx*	GU937429	TCTGCTATCATTCGCGGACT	102
			CTTGGTCGGCATACTGGTTC	

* Upper and lower sequences represent forward and reverse primers, respectively.

### Preparation of samples for the detection of MT, GSH/GSSG, 4-OHE_2_/E_2_ and E_2_/(E_2_)-3,4-Q

The *E*. *fetida* were frozen in liquid nitrogen and disrupted. The frozen samples were further homogenized using an Ultra-Turrax T8 homogenizer (IKA, Staufen, Germany). Subsequently, 1 mL of 0.2 M phosphate buffer (p*H* = 7.0) was added, and the samples were homogenized for another 5 min. The homogenates were centrifuged (16,000 × *g*, 15 min, 4°C) using an Eppendorf 5402 microcentrifuge (Eppendorf, Hamburg, Germany). The supernatant was further filtered through a membrane filter (0.45-μm nylon filter disk, Millipore, Billerica, MA, USA).

The 4-OHE_2_/E_2_ and E_2_/(E_2_)-3,4-Q ratio was determined in the *E*. *fetida* homogenates using a matrix-MALDI-TOF/TOF mass spectrometer (Bruker ultrafleXtreme (Bruker Daltonik, GmbH, Bremen, Germany). The matrix used for the analyses was α-cyano-4-hydroxycinnamic acid (HCCA) with the addition of ammonium citrate (0.8 mg in 100 μL of water). The spectra were typically acquired from the average of 20 subspectra from a total of 500 shots of the laser.

Prior to the analysis of glutathiones and phytochelatins, the samples were enriched with 10% trifluoroacetic acid (ratio 1:1). Subsequently, the mixtures were centrifuged (4°C, 25,000 × *g*, 20 min) using an Eppendorf 5402 microcentrifuge, and the supernatant was employed for HPLC-ED measurements.

For the determination of metallothionein, 1 mL of sample was denatured at 99°C for 20 min in a Thermomixer comfort (Eppendorf, Hamburg, Germany) and centrifuged for 10 min (Eppendorf 5402 microcentrifuge) to remove high-abundance proteins and peptides.

### Quantification of free radicals and total protein content

Free radicals were quantified using the chlorophyllin assay (Free Radicals kit, Sevapharma, Prague, Czech Republic) as previously described [40]. The total protein content was determined for standardization, performed using a SKALAB CBT 600T kit (Skalab, Svitavy, Czech Republic) according to the manufacturer's instructions. For both analyses, a BS-400 automated spectrophotometer (Mindray, Schenzhen, China) was employed.

### Determination of metallothionein

MT was electrochemically quantified using differential pulse voltammetry (DPV) as previously described [41].

### Determination of GPx enzymatic activity

A Glutathione Peroxidase Cellular Activity Assay Kit (CGP1, Sigma-Aldrich) was employed to determine GPx enzymatic activity. The analyses were performed according to the manufacturer's instructions using a BS-400 automated spectrophotometer (Mindray).

### HPLC-ED of phytochelatins and glutathiones

The antioxidant molecule contents were determined using high performance liquid chromatography coupled with an electrochemical detector (HPLC-ED) as previously described for the analyses of GSH/GSSG [42] and PCs [43].

### Histological procedure

The samples were fixed in formaldehyde (10%) overnight, subsequently dehydrated in serial ethanol concentrations and embedded in paraffin wax. The sections were cut at 5 μm, mounted onto glass slides, deparaffinized and stained with haematoxylin-eosin. Microscopic observations were performed using an Olympus DP73 microscope (Olympus, Tokyo, Japan).

### Matrix-assisted laser desorption/ionization imaging mass spectrometry

*E*. *fetida* sections were mounted onto ITO glass slides (Bruker Daltonik, GmbH), and the slides were scanned using an Epson Perfection V500 scanner (Epson Europe, Amsterdam, Netherland) with a resolution of 2400 DPI. Subsequently, HCCA was prepared (7 mg/mL in 50% acetonitrile and 0.2% trifluoroacetic acid) and sprayed using ImagePrep (Bruker Daltonik, GmbH).

The mass spectrometry experiments were performed on a MALDI-TOF/TOF Bruker ultrafleXtreme mass spectrometer (Bruker Daltonik, GmbH). The scanning raster was set to 50 μm, and prior to each measurement, the mass spectrometer was calibrated using a standard calibration mixture of proteins and peptides (Bruker). For low-mass molecules, imaging mass spectrometry (IMS) was performed in reflectron positive mode, and for metallothioneins the IMS was performed in linear positive mode; both analyses were performed at 45% laser power. The MS spectra were typically acquired after averaging 500 subspectra from a total of 500 laser shots per raster spot. After analyses, the mass spectra were automatically loaded into flexAnalysis and subsequently processed (baseline subtraction).

Moreover, IMS figures were prepared after selecting the peaks of interest—PC_2_/PC_3_, (E_2_)-3,4-Q, GSSG and MTs. The molecular weights were selected according to the UniProt database (www.uniprot.org) and processed in a molecular weight + hydrogen ± 0.05% format.

### Descriptive statistics

The mathematical analysis of the data and graphical interpretations were performed using Microsoft Excel^®^, Microsoft Word^®^ and Microsoft PowerPoint^®^. The results are expressed as the mean ± standard deviation unless otherwise noted. Differences between the groups were analysed using paired t-test and ANOVA. Unless noted otherwise, the threshold for significance was *p* <0.05. Statistica 12 software (StatSoft, Tulsa, OK, USA) was employed for all analyses.

## Conclusion

The results of the present study demonstrated that exposure to E_2_ might affect *E*. *fetida* with respect to oxidative stress. A significant impact on fundamental ecotoxicological parameters, such as the reproduction rate and biomass production, was observed. The analysis of gene expression and identification of antioxidant molecules revealed that E_2_ generates oxidative stress in the cellular microenvironment of *E*. *fetida* through antioxidant defence systems, comprising glutathiones and metallothioneins. These mechanisms protect the earthworm against the harmful effects of hormones. These results also showed the presence of three various isoforms of PCS in *E*. *fetida*; however, exposure to E_2_ decreased the tissue concentration of PCS, reflecting the oxidation of GSH to GSSG. In conclusion, E_2_ generated oxidative stress in cells, which do not contain oestrogen receptors, through bioaccumulation and further degradation to redox cycling forms. To further elucidate this phenomenon, metabolomics studies describing the interactions between E_2_ and enzyme antioxidants (CAT, SOD) in *E*. *fetida* could be performed together with experiments utilizing actual complex soil, where the soil structure and/or other soil biota affect the bioaccumulation and degradation of E_2_. Understanding the effects of pollutants from EDCs on soil inhabitants, particularly earthworms, could enhance the potential utilization of these organisms as biomarkers of environmental status.
